# ID 257 - Comparação de Estimativas de Pacientes com Esclerose Múltipla Elegíveis para o Uso de Fingolimode no Sistema Único de Saúde (SUS) e Dados de Mundo Real

**DOI:** 10.5327/2237-9622.2025.v34s1.257

**Published:** 2025-11-25

**Authors:** Renata Cristina Ferreira Rola, Alef Cleto Almeida, Nathalia Volpi e Silva, Daniela Atique Vicentini, Aline Mauch dos Santos, Aline Gomes Rocha, Júlia Lima, Pedro Antônio Guimarães Notaroberto Barbosa, Renato Mantelli Picoli

**Keywords:** esclerose múltipla, fingolimode, SUS, pós-incorporação de tecnologia

## Abstract

**Introdução:**

Uma das etapas para a incorporação de tecnologia no SUS é a estimativa de pacientes elegíveis para receber a tecnologia, considerando um horizonte temporal de cinco anos. Em vista disso, o presente estudo visa comparar as estimativas de pacientes com esclerose múltipla remitente recorrente (EMRR) elegíveis para o uso de fingolimode no SUS apresentadas no relatório de recomendação com dados de mundo real, a fim de verificar se, no período pós-incorporação, as previsões aprovadas foram efetivamente cumpridas e se estão condizentes com a realidade do sistema público brasileiro.

**Método:**

Os dados utilizados como referência para a análise foram extraídos do relatório de recomendação final da Comissão Nacional de Incorporação de Tecnologias no SUS (Conitec) n.º 113, publicado no ano de 2014, em relação ao uso de fingolimode para tratamento da EMRR. Os dados de interesse foram as estimativas e a distribuição da população elegível ao fingolimode e seus comparadores () no horizonte temporal de 2014 a 2018. Para efeitos comparativos, foi extraído do Departamento de Informação e Informática do SUS (DataSUS) todo o histórico de pacientes diagnosticados com CID G35 no período entre 2014 e 2020, considerando pacientes que iniciaram o tratamento de primeira linha a partir de 2014. Para estabelecer a população com EMRR no SUS, foi empregada a mesma porcentagem utilizada pelo demandante no relatório de recomendação de que 85% dos indivíduos com EM possuem EMRR.

**Resultados:**

A população estimada pelo demandante no relatório de recomendação que possuía EMRR tratada no SUS foi menor do que a encontrada nos dados de vida real para o mesmo período analisado. A subestimativa dessa população variou entre 20% e 37% no horizonte temporal de 2014-2018. A taxa de difusão proposta no relatório de recomendação para o, para pacientes com EMRR em uso de primeira de linha de tratamento, foi de 8,5% para o ano de 2014, com um incremento de 5,5%, 1,5%, 1,0% e 0,5% para os anos seguintes. Contudo, com base nos dados do DataSUS, foi observado que esses valores foram superestimados, isso porque a utilização do fingolimode consta somente a partir de 2015 e, somado a isso, nesse ano, o alcance da tecnologia foi metade do estimado no relatório de recomendação. No período de 2016-2018, o incremento cumulativo na taxa de difusão foi de 4,4% (variando de 0,3% a 1,9% ao ano). Ademais, em análise complementar ao horizonte temporal, foi verificado que a proporção de pacientes proposta para o ano de 2017 foi atingida apenas em 2020.

**Conclusão:**

Os resultados indicam uma diferença relevante entre a população elegível para fingolimode avaliada no relatório de recomendação quando comparada aos pacientes encontrados no DataSUS, demonstrando, com isso, que a estimativa da população elegível é um desafio devido à ausência de dados epidemiológicos de alta especificidade (como os pacientes com EMRR) e generalizáveis para toda a população brasileira. Já o cumprimento da taxa de difusão proposta no relatório de recomendação pode ser influenciado por outros fatores externos, como menor adoção da tecnologia pelos serviços, dificuldades na distribuição e financiamento. Esses dados reforçam que uma estimativa adequada da população elegível é de suma importância porque impacta principalmente a previsibilidade do orçamento a ser destinado para o tratamento de uma doença específica, além de promover e garantir o acesso da população às novas tecnologias incorporadas no sistema público de saúde brasileiro.

